# In vivo evaluation of synthetic cannabinoid JWH-018 derivatives as potential new psychoactive substances

**DOI:** 10.1038/s41598-026-51639-z

**Published:** 2026-05-11

**Authors:** Salman Khan, Minseo Baek, Jeong-Hoon Jang, In-Soo Myeong

**Affiliations:** College of Pharmacy, Daegu Catholic University, Gyeongsan, Gyeongbuk 38430 Republic of Korea

**Keywords:** Drug discovery, Medical research, Neuroscience

## Abstract

Synthetic cannabinoid receptor agonists constitute a prominent class of new psychoactive substances (NPS), in which minor structural modifications can markedly influence in vivo pharmacological outcomes. In this study, methoxy-substituted derivatives of **JWH-018** were synthesized to examine the effect of substitution position on behavioral activity. **JWH-018–5-OMe** and **JWH-018–6-OMe** were evaluated using four in vivo assays, including catalepsy, hypothermia, analgesia, and locomotor activity. JWH-018–5-OMe selectively reduced rectal temperature, with limited effects observed in catalepsy, analgesia, and locomotor activity. Notably, **JWH-018–6-OMe** elicited robust and dose-dependent effects across all four behavioral endpoints, closely resembling the pharmacological profile of the parent compound, **JWH-018**. Collectively, these results demonstrate that structural variations on the indole scaffold can produce measurable psychoactive effects, underscoring the importance of continued evaluation of indole-based synthetic cannabinoids within the NPS framework.

**Supplementary Information:**

The online version contains supplementary material available at 10.1038/s41598-026-51639-z.

## Introduction

New psychoactive substances (NPS) represent a major and continuously evolving public health challenge worldwide^1^. Their rapid structural diversification, ease of dissemination, and frequent circumvention of existing legal controls have resulted in significant societal harm, including outbreaks of acute intoxication, unpredictable toxicological effects, and an increasing burden on emergency medical and forensic systems^2,3^. Among NPS, synthetic cannabinoid receptor agonists (SCRAs)—often marketed as “synthetic marijuana” under brand names such as K2 or Spice—have emerged as one of the most problematic classes, owing to their exceptionally high potency, inconsistent chemical composition, and limited detectability in routine drug screening^4–6^. Notably, the overall risk posed by NPS continues to escalate as newly designed analogues and formulations are introduced, enabling persistent evasion of regulatory frameworks and further complicating surveillance and enforcement efforts^2,7,8^.

Within this broader context, the emergence of **JWH-018** (**1**) and related naphthoylindole-based derivatives represents an early and influential example of how SCRAs rapidly disrupted both public health and regulatory systems. These compounds were widely incorporated into commercial “K2/Spice” products, driving extensive recreational use while simultaneously generating substantial uncertainty regarding their legal status and toxicological risk profiles^4,6,9^. Collectively, these outcomes indicate that **JWH-018**-type SCRAs were not merely early members of the class, but catalysts for a persistent pattern of harm across subsequent generations of synthetic cannabinoids^2,4,6^.

Notably, this phenomenon has not been limited to Western countries. In South Korea, recent forensic and analytical evidence indicates a sustained increase in the detection of new NPS, including synthetic cannabinoids and their structural analogues. National forensic data show that both the number and structural diversity of newly identified psychoactive substances have risen steadily in recent years, with synthetic cannabinoids accounting for a substantial proportion of compounds detected in seized materials and biological specimens^10^. These findings further indicate that many of the detected substances are newly designed analogues intended to evade existing scheduling controls, reflecting a regulatory “cat-and-mouse” dynamic similar to that observed internationally. As a result, despite historically low levels of illicit drug use, the rapid emergence and diversification of NPS now represent a growing challenge for public health, law enforcement, and forensic systems in South Korea.

Taken together, these observations show that, although first-generation synthetic cannabinoids such as **JWH-018** (**1**) may decline after legal control, the broader threat persists through continued structural modification and market adaptation^11,12^. To help address this ongoing public-health concern, the present study focuses on the synthesis and in vivo evaluation of **JWH-018** (**1**) derivatives with the potential to function as new psychoactive substances, providing pharmacological and toxicological insight relevant to early risk assessment and regulatory consideration^12,13^.

**Fig. 1:**
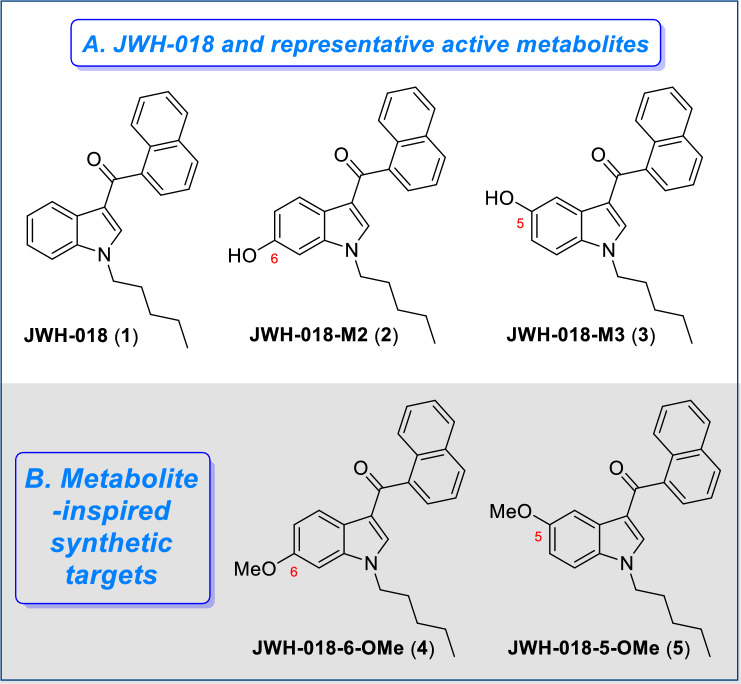
Structures of **JWH-018**, representative active metabolites, and synthetic targets in this study. Chemical structures of **JWH-018**, its representative active metabolites, and the metabolite-inspired synthetic targets investigated in this study.

For **JWH-018** (**1**), hydroxylation on the aromatic indole ring has been identified as a major metabolic pathway, and several of these hydroxylated metabolites have been reported to retain significant pharmacological activity. In particular, metabolites bearing a hydroxyl group at the 5- or 6-position of the indole moiety have been shown to exhibit substantial cannabinoid receptor activity, highlighting their potential relevance to the in vivo effects of **JWH-018** (**1**) (Fig. 1)^13,14^. Motivated by these observations, we focused on these metabolically relevant positions and synthesized compounds **4** and **5**, bearing methoxy substituents at the 6- and 5-positions, respectively.

Given the structural relevance of these positions, it is essential to determine how such modifications influence the overall pharmacological impact in vivo. To this end, we evaluated the pharmacological profiles of these metabolite-inspired derivatives using the cannabinoid tetrad assay. This well-established behavioral model that assesses four key parameters- catalepsy, hypothermia, antinociception, and suppressed locomotor activity- and is widely used to characterize the behavioral effects of *CB*_*1*_ receptor agonists in rodents^15–17^. By assessing these four distinct parameters, we aimed to provide a comprehensive physiological and behavioral evaluation, enabling a systematic comparison between these novel analogues and established cannabinoids like **JWH-018** (**1**). This approach is instrumental for the preliminary risk assessment of emerging SCRAs, as it correlates specific structural modifications with their subsequent biological outcomes. Through this comparative analysis, we aimed to determine whether metabolite-inspired modifications of the indole ring give rise to compounds with potent in vivo activity, thereby providing critical insights into the risk profiles of potential NPS.

## Results

### Synthesis of JWH-018–6-OMe (4) and JWH-018–5-OMe (5)

**Fig. 2:**
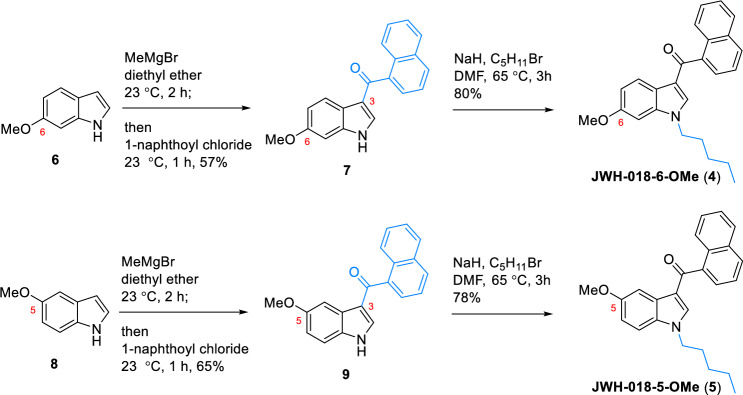
Synthesis of **JWH-018–6-OMe** (**4**) and **JWH-018–5-OMe** (**5**). Synthetic routes to **JWH-018–6-OMe** (**4**) and **JWH-018–5-OMe** (**5**) via acylation of the corresponding indoles followed by *N*-alkylation.

The synthetic routes to **JWH-018–6-OMe** (**4**) and **JWH-018–5-OMe** (**5**) are outlined in Fig. 2. Acylation of the corresponding methoxy-substituted indoles **6** and **8** with 1-naphthoyl chloride afforded the ketone intermediates **7** and **9** in 57% and 65% yield, respectively. To gain insight into the mechanistic features of the C-3 acylation of indoles, a widely accepted mechanism suggests abstraction of *N*–H proton by highly basic Grignard reagent to generate the corresponding indolyl Grignard intermediate. This Grignard intermediate subsequently enhances the nucleophilicity of the already nucleophilic C-3 carbon, which in turn reacts with the 1-naphthoyl chloride to yield the corresponding 3-acylated indole derivative^18,19^. Subsequent *N*-alkylation of intermediates **7** and **9** was achieved using NaH and 1-bromopentane in DMF, providing the target compounds **JWH-018–6-OMe** (**4**) and **JWH-018–5-OMe** (**5**) in 80% and 78% yield, respectively^18^. This straightforward two-step sequence enabled efficient access to both methoxy-substituted **JWH-018** (**1**) derivatives for further biological evaluation.

### In vivo evaluation of JWH-018–5-OMe (5)

**Fig. 3:**
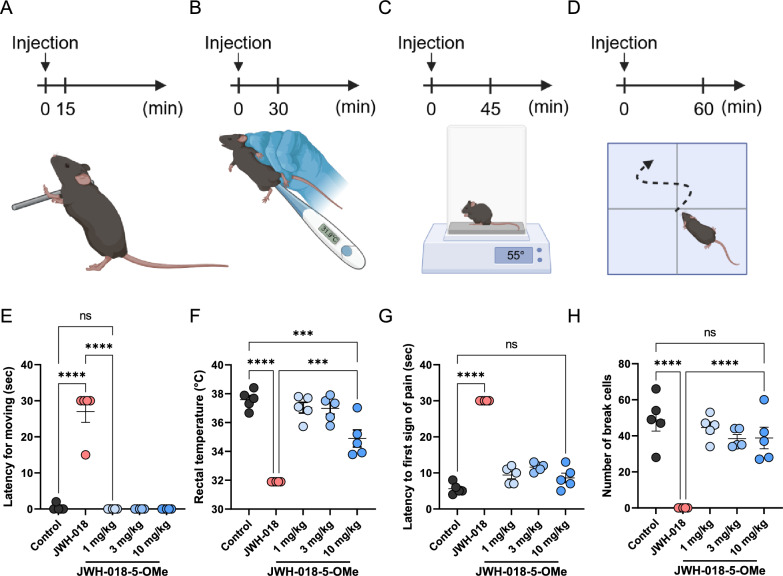
Evaluation of cannabinoid-like effects of **JWH-018–5-OMe** in the mouse tetrad assay. The pharmacological effects of **JWH-018–5-OMe** were assessed at various doses (1, 3, and 10 mg/kg, i.p.) and compared with the vehicle control and positive control (**JWH-018**, 3 mg/kg, i.p.). (**A**–**D**) Schematic representation of the tetrad assay time course: (**A**) catalepsy, (**B**) hypothermia, (**C**) analgesia, and (**D**) locomotor activity. (**E**) Catalepsy assessment measured by the bar test. (**F**) Rectal temperature changes. (**G**) Antinociceptive effects measured by the hot plate test. (**H**) Spontaneous locomotor activity measured by the number of grid crossings in an open-field test. Data are presented as mean ± SEM (n = 5 per group). ****p < 0.0001, ***p < 0.001 vs. vehicle control; ns, non-significant.

The pharmacological profile of **JWH-018–5-OMe** (**5**) was evaluated using the cannabinoid tetrad assay—comprising catalepsy, hypothermia, analgesia, and locomotor activity—to determine its potential psychoactive effects relative to the established synthetic cannabinoid **JWH-018** (**1**, Fig. 3A–D). The assessment time points (15, 30, 45, and 60 min post-injection) were specifically timed to coincide with the peak brain concentrations (C*max*) and maximal behavioral effects typically observed within one hour for indole-derived cannabinoids^20^.

In the bar test for catalepsy and the hot plate test for antinociceptive activity, the positive control **JWH-018** (**1**) exhibited profound cataleptic and analgesic responses compared to the vehicle control group (Fig. 3E,G). In contrast, **JWH-018–5-OMe** (**5**) did not show any significant cataleptic or analgesic responses across all tested doses (Fig. 3E,G). Similarly, **JWH-018–5-OMe** (**5**) did not significantly alter spontaneous locomotor activity, with the number of grid crossings remaining comparable to the control group (Fig. 3H). However, it is particularly interesting to note that despite the absence of these classical behavioral markers, **JWH-018–5-OMe** (**5**) induced a significant and selective reduction in rectal temperature, especially at the 10 mg/kg dose (*p* < 0.001) (Fig. 3F). This distinct divergence from the typical cannabinoid tetrad profile suggests a unique pharmacological interaction of the 5-methoxy derivative.

### In vivo evaluation of JWH-018–6-OMe (4)

**Fig. 4:**
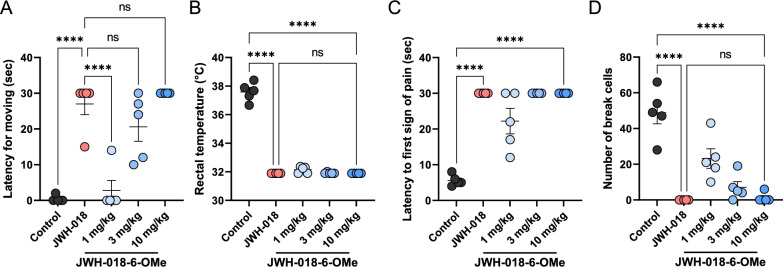
Evaluation of cannabinoid-like effects of **JWH-018–6-OMe** in the mouse tetrad assay. The pharmacological effects of **JWH-018–6-OMe** were evaluated for its potent cannabinoid-like behavioral activity. (**A**) Catalepsy assessment. (**B**) Hypothermia assessment. (**C**) Analgesia assessment. (**D**) Locomotor activity. Data are presented as mean ± SEM. ****p < 0.0001 vs. vehicle control; ns, non-significant.

**Fig. 5:**
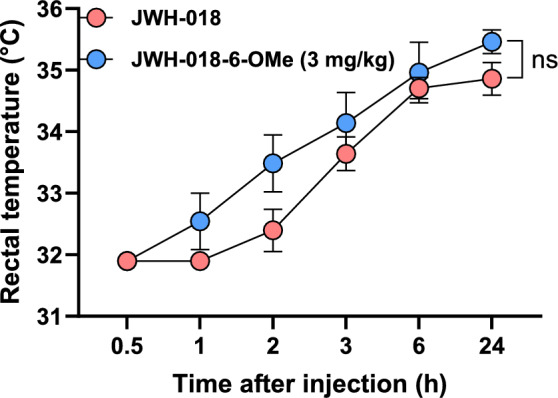
Time-course recovery of rectal temperature following **JWH-018–6-OMe** administration. The duration of action and recovery profile of **JWH-018–6-OMe** (3 mg/kg, i.p.) were compared with the positive control **JWH-018** (3 mg/kg, i.p.) over 24 h. Rectal temperatures were monitored at indicated time points post-injection. Data are expressed as mean ± SEM (n = 5). ns, non-significant.

In striking contrast to the 5-methoxy derivative, **JWH-018–6-OMe** (**4**) exhibited a potent pharmacological profile that was almost identical to that of the positive control, **JWH-018** (**1**). Administration of **JWH-018–6-OMe** (**4**) resulted in a robust, dose-dependent increase in the latency for moving during the bar test, with the 10 mg/kg dose inducing cataleptic effects that were statistically indistinguishable from those of **JWH-018** (**1**, Fig. 4A). Furthermore, all tested doses of **JWH-018–6-OMe** (**4**) triggered a rapid and profound drop in rectal temperature, reaching approximately 32 °C, which mirrors the severe hypothermia observed in the **JWH-018** (**1**) group (Fig. 4B). The compound also demonstrated significant antinociceptive activity; mice treated with 3 and 10 mg/kg doses reached the maximum cut-off time (30 s) in the hot plate test, reflecting a complete suppression of thermal pain response identical to the effects of the scheduled drug (Fig. 4C). Additionally, spontaneous locomotor activity was markedly reduced in a dose-dependent manner, confirming the potent immobilizing and sedative properties of this derivative (Fig. 4D). Notably, time-course analysis revealed that the recovery trajectory of rectal temperature for **JWH-018–6-OMe** (**4**, 3 mg/kg) closely followed that of JWH-018 **(1)**, with a gradual return toward baseline over a 24-h period, further highlighting their pharmacological equivalence (Fig. 5). Taken together, these results provide definitive evidence that **JWH-018–6-OMe** (**4**) possesses a cannabinoid-like behavioral similarity comparable to that of **JWH-018** (**1**), fulfilling all the classical behavioral hallmarks of potent cannabinoid agonists.

## Discussion

The results of this study provide critical insights into the structure–activity relationship (SAR) of **JWH-018** (**1**) derivatives, demonstrating that the specific position of methoxy substitution on the indole scaffold is a primary determinant of their in vivo pharmacological potency. Notably, **JWH-018–6-OMe** (**4**) exhibited a comprehensive “tetrad” profile that closely mirrors the effects of the parent compound, **JWH-018** (**1**), whereas **JWH-018–5-OMe** (**5**) failed to elicit a tetrad response substantially comparable to that of **JWH-018 (1)**.

In the catalepsy assessment, the robust immobility induced by **JWH-018–6-OMe** (**4**) suggests potent central nervous system (CNS) depression mediated by the activation of CB₁ receptors within the basal ganglia^21^. This effect is consistent with established pharmacological models where CB₁ agonists modulate dopaminergic and GABAergic neurotransmission in the striatum to inhibit motor execution^21–24^. Interestingly, while **JWH-018–5-OMe** (**5**) failed to induce significant catalepsy, it did maintain a significant hypothermic effect. This selective reduction in rectal temperature indicates that both derivatives preserve the typical synthetic cannabinoid profile regarding thermoregulation, likely through CB₁ receptor-mediated actions in the preoptic area of the hypothalamus^25–29^. The fact that the 5-OMe derivative retains its hypothermic action despite losing other tetrad effects suggests that thermoregulatory circuits may be more sensitive to structural variations or represent a distinct signaling efficiency compared to other behavioral endpoints^30^.

The antinociceptive potential of these compounds, evaluated through the hot plate test, further reinforces the position-dependent activity of the methoxy group. **JWH-018–6-OMe** (**4**) produced a significant increase in pain latency, indicating that CB₁ receptor activation in the periaqueductal gray (PAG) and spinal cord—key regions for modulating supraspinal and spinal pain pathways—remains highly effective^31–34^. This potent analgesia, coupled with the marked hypolocomotion observed in the open-field test, confirms that the 6-OMe substitution preserves the high-potency psychotropic profile characteristic of synthetic cannabinoids^17^. The observed decrease in spontaneous locomotor activity is a hallmark of CB₁-mediated CNS inhibition, a phenomenon that has been shown to be entirely absent in CB₁-knockout models^17,35^.

In this study, female mice were exclusively used to evaluate the pharmacological potential of JWH-018 derivatives, as females have been reported to exhibit higher sensitivity to the behavioral effects of cannabinoids, often attributed to higher levels of active metabolites compared to males. While we did not monitor the estrous cycle in this study, prior research suggests that the acute and robust pharmacological responses in the cannabinoid tetrad assay are primarily driven by dosage and the route of administration, rather than subtle hormonal fluctuations associated with the estrous cycle^36^. Nevertheless, future studies incorporating both sexes and considering hormonal status will be beneficial to further elucidate the sex-dependent pharmacokinetics of these novel analogues.

In conclusion, **JWH-018–6-OMe** (**4**) exhibits a psychoactive profile closely resembling that of **JWH-018** (**1**), suggesting that it may be classified as a potential NPS. Given its comparable in vivo behavioral effects, this compound warrants early surveillance and proactive regulatory consideration, demonstrating that structural variations on the indole scaffold can produce measurable psychoactive effects.

## Materials and methods

### Chemical synthesis

#### General information

All reactions were performed in oven-dried round-bottom flasks fitted with rubber septa and were conducted under positive nitrogen pressure, unless noted otherwise. Gas-tight syringes with stainless steel needles were used to transfer all liquids. Flash column chromatography was performed as described by Still et al.^37^ using granular silica gel 60 (Merck 0.040–0.063 mm). Analytical thin layer chromatography (TLC) was performed using glass plates pre-coated with 0.25 mm 230–400 mesh silica gel impregnated with a fluorescent indicator (254 nm). TLC plates were visualized by exposure to short wave ultraviolet light (254 nm) and irreversibly stained by treatment with an aqueous solution of ceric ammonium molybdate (CAM) (~ 1 min) on a hot plate. Organic solutions were concentrated at 40 °C on rotary evaporators capable of achieving a minimum pressure of ~ 10 Torr.

### Materials

Commercial reagents and solvents were used as received with the following exceptions: diethyl ether and *N,N*-dimethylformamide were purchased from Samchun chemicals and TCI South Korea respectively and were stored over pre-activated 4 Å molecular sieves and were used under positive nitrogen pressure. Deuterated solvents used for nuclear magnetic resonance (NMR) spectroscopy were purchased from Cambridge Isotope Laboratories, Inc. and were used as received. 5-methoxy indole was purchased from Fluka, 1-bromopentane and 1-naphthoyl chloride were purchased from Alfa Aesar, whereas 6-methoxy indole and NaH (60% in mineral oil) were purchased from TCI chemicals South Korea.

### Instrumentation

Proton nuclear magnetic resonance (^1^H NMR) spectra were recorded with a Bruker AVANCE NEO 600 spectrometer. Spectra were processed with Bruker Topspin 4.5.0 using the automatic phasing and baseline correction for data up to 6D. Chemical shifts are recorded in parts per million on the δ scale and are referenced from the residual protium in the NMR solvent (CHCl_3_: δ 7.26). Data are reported as follows: chemical shift [multiplicity (s = singlet, d = doublet, t = triplet, q = quartet, m = multiplet, br = broad), coupling constant(s) in Hertz, integration, assignment]. Apparent multiplets that correspond to two distinct signals in proximity are annotated as “app”. Carbon-13 nuclear magnetic resonance (^13^C NMR) spectra were recorded with a Bruker AVANCE NEO 600 spectrometer and are recorded in parts per million on the δ scale and are referenced from the carbon resonances of the solvent (CDCl_3_: δ 77.16). Data are reported as follows: chemical shift (assignment). High-resolution mass spectra (HRMS) were recorded on a JEOL JMS-700 mass spectrometer equipped with a magnetic sector analyzer, using electron ionization (EI) mode. The purity of all synthesized compounds was confirmed to be ≥ 95% using a Waters 2695 HPLC system (Waters Corporation, Milford, MA, USA) equipped with a Waters 2487 Dual λ Absorbance detector. For chromatographic separation, an Agilent Eclipse Plus C18 column (250 × 4.6 mm) was utilized at a column temperature of 30 ℃. The mobile phases consisted of water containing 0.1% TFA (v/v) (phase A) and acetonitrile (phase B), with a flow rate set at 1.0 mL/min. The gradient elution program was as follows: 0 min A: 30%, B: 70%; 30 min A: 10%, B: 90%; 36 min A: 10%, B: 90%; 37 min A: 30%, B: 70%; 45 min A: 30%, B: 70%.

Compounds **1**, **4**, **5**, **7** and **9** were synthesized according to literature procedure described by Makriyannis et al.^38^.

#### Compound 1

Compound **1** was synthesized according to a literature procedure^38^.

*R*_f_ = 0.7 (30% EtOAc in hexanes); ^1^H NMR (600 MHz, CDCl_3_) *δ* (ppm) 8.49–8.48 (m, 1H, Ar**H**), 8.19 (d, *J* = 8.39 Hz, 1H, Ar**H**), 7.97 (d, *J* = 8.25 Hz, 1H, Ar**H**), 7.91 (d, *J* = 8.07 Hz, 1H, Ar**H**), 7.66 (dd, *J* = 6.90, 0.86 Hz, 1H, Ar**H**), 7.54–7.50 (m, 2H, Ar**H**), 7.48–7.46 (m, 1H, Ar**H**), 7.41–7.35 (m, 4H, Ar**H**), 4.07 (t, *J* = 7.44 Hz, 2H, -NC**H**_**2**_-), 1.81 (quint, *J* = 7.51 Hz, 2H, -NCH_2_C**H**_**2**_CH_2_-), 1.34–1.23 (m, 4H, -C**H**_**2**_C**H**_**2**_CH_3_), 0.85 (t, *J* = 6.93 Hz, 3H, -CH_2_C**H**_**3**_) ppm; ^13^C NMR (150.9 MHz, CDCl_3_, 25 °C): *δ* 192*.*03 (-**C** = O)*,* 139.16 (Ar), 138.02 (Ar), 137.09 (Ar), 133.79 (Ar), 130.85 (Ar), 130.00 (Ar), 128.23 (Ar), 127.06 (Ar), 126.78 (Ar), 126.32 (Ar), 126.03 (Ar), 125.88 (Ar), 124.62 (Ar), 123.64 (Ar), 122.94 (Ar), 122.87 (Ar), 117.54 (Ar), 110.09 (Ar), 47.16 (-N**C**H_2_-), 29.50 (-NCH_2_**C**H_2_-), 28.91 (-CH_2_**C**H_2_CH_2_-), 22.20 (-**C**H_2_CH_3_), 13.92 (-CH_2_**C**H_3_) ppm. HRMS (EI) calcd for C_24_H_23_NO [M]⁺· 341.178, found 341.1779. HPLC purity: 99.52%; retention time: 13.8 min.

#### Compound 7

To a stirred solution of 6-methoxy indole **6** (200 mg, 1.36 mmol) in dry diethyl ether 15 mL was added MeMgBr dropwise (3 M in diethyl ether, 0.5 mL, 1.5 mmol, 1.1 equiv) at 0 °C. Then the reaction was stirred at 23 °C for 2 h followed by dropwise addition of 1-naphthoyl chloride (0.2 mL, 1.36 mmol, 1.0 equiv) dissolved in dry diethyl ether (5 mL) at 0 °C. The resulting solution was then stirred at 23 °C for additional 1 h. The reaction was then quenched with sat. NH_4_Cl (15 mL) and stirred for 10 min followed by addition of 20 mL H_2_O and extraction with EtOAc (3 × 25 mL). Combined organics were dried over MgSO_4_, solvent evaporated under vacuo and chromatographed to give compound **7** (233 mg, 57%) as an off white solid.

*R*_f_ = 0.5 (30% EtOAc in hexanes); ^1^H NMR (600 MHz, CDCl_3_, 25 °C) *δ* 8.56 (br. s, 1H, N**H**), 8.37 (d, *J* = 8.77 Hz, 1H, Ar**H**), 8.17 (d, *J* = 8.31 Hz, 1H, Ar**H**), 7.95 (d, *J* = 8.23 Hz, 1H, Ar**H**), 7.89 (d, *J* = 7.98 Hz, 1H, Ar**H**), 7.64 (dd, *J* = 6.92, 0.83 Hz, 1H, Ar**H**), 7.50 (t, *J* = 7.07 Hz, 2H, Ar**H**), 7.46 (td, *J* = 8.49, 1.35 Hz, 1H, Ar**H**), 7.30 (d, *J* = 2.98 Hz, 1H, Ar**H**), 7.01 (dd, *J* = 8.71, 2.25 Hz, 1H, Ar**H**), 6.90 (d, *J* = 2.96 Hz, 1H, Ar**H**), 3.89 (s, 3H, -OC**H**_3_) ppm; ^13^C NMR (150.9 MHz, CDCl_3_, 25 °C): *δ* 193.00 (-**C** = O), 156.79 (Ar), 138.92 (Ar), 135.42 (Ar), 133.84 (Ar), 131.43 (Ar), 130.84 (Ar), 130.17 (Ar), 128.32 (Ar), 126.94 (Ar), 126.45 (Ar), 125.97 (Ar), 124.64 (Ar), 119.00 (Ar), 114.80 (Ar), 112.45 (Ar), 103.77 (Ar), 55.94 (-O**C**H_3_) ppm. HRMS (EI) calcd for C_20_H_15_NO_2_ [M]⁺· 301.1103, found 301.1100.

#### Compound 9

To a stirred solution of 5-methoxy indole **8** (200 mg, 1.36 mmol) in dry diethyl ether 15 mL was added MeMgBr dropwise (3 M in diethyl ether, 0.5 mL, 1.5 mmol, 1.1 equiv) at 0 °C. Then the reaction was stirred at 23 °C for 2 h followed by dropwise addition of 1-naphthoyl chloride (0.2 mL, 1.36 mmol, 1.0 equiv) dissolved in dry diethyl ether (5 mL) at 0 °C. The resulting solution was then stirred at 23 °C for additional 1 h. The reaction was then quenched with sat. NH_4_Cl (15 mL) and stirred for 10 min followed by addition of 20 mL H_2_O and extraction with EtOAc (3 × 25 mL). Combined organics were dried over MgSO_4_, solvent evaporated under vacuo and chromatographed to give compound **9** (225 mg, 65%) as an off pale-yellow solid.

*R*_f_ = 0.6 (30% EtOAc in hexanes); ^1^H NMR (600 MHz, CDCl_3_) *δ* 8.66 (br. s, 1H, N**H**), 8.17 (d, *J* = 8.40 Hz, 1H, Ar**H**), 8.02 (s, 1H, Ar**H**), 7.95 (d, *J* = 8.24 Hz, 1H, Ar**H**), 7.89 (d, *J* = 8.14 Hz, 1H, Ar**H**), 7.64 (d, *J* = 6.86 Hz, 1H, Ar**H**), 7.50 (t, *J* = 8.14 Hz, 2H, Ar**H**), 7.46 (t, *J* = 7.33 Hz, 1H, Ar**H**), 7.34 (d, *J* = 2.92 Hz, 1H, Ar**H**), 7.31 (d, *J* = 8.77 Hz, 1H, Ar**H**), 6.98 (dd, *J *= 8.81, 2.15 Hz, 1H, Ar**H**), 3.92 (s, 3H, -OCH^3^) ppm; ^13^C NMR (150.9 MHz, CDCl_3_, 25 °C): *δ* 192.76 (-**C** = O), 157.78 (Ar), 138.85 (Ar), 137.60 (Ar), 134.24 (Ar), 133.85 (Ar), 130.89 (Ar), 130.23 (Ar), 128.31 (Ar), 126.95 (Ar), 126.43 (Ar), 126.07 (Ar), 126.02 (Ar), 124.60 (Ar), 123.45 (Ar), 120.20 (Ar), 119.38 (Ar), 112.50 (Ar), 95.17 (Ar), 55.82 (-O**C**H_3_) ppm.  HRMS (EI) calcd for C_20_H_15_NO_2_ [M]⁺· 301.1103, found 301.1101.

#### Compound 4

To a solution of NaH (52 mg, 1.30 mmol, 3.0 equiv, 60% in mineral oil) in dry DMF 5 mL, was added compound **7** (130 mg, 0.432 mmol) and the resulting solution was stirred at rt for 30 min. Then, 1-bromopentane (80 *µ*L, 0.65 mmol, 1.5 equiv) was added and the resulting solution was stirred at 65 °C for 3 h. The reaction was then quenched with sat. NH_4_Cl (10 mL) and stirred for 20 min and extracted with EtOAc (3 × 20 mL). Combined organics were dried over MgSO_4_, solvent evaporated under vacuo and chromatographed to give compound **4** (128 mg, 80%) as a colourless waxy solid.

*R*_f_ = 0.4 (20% EtOAc in hexanes); ^1^H NMR (600 MHz, CDCl_3_) *δ* (ppm) 8.37 (d, *J* = 8.78 Hz, 1H, Ar**H**), 8.19 (d, *J* = 8.42 Hz, 1H, Ar**H**), 7.96 (d, *J* = 8.27 Hz, 1H, Ar**H**), 7.91 (d, *J* = 8.1 Hz, 1H, Ar**H**), 7.65 (d, *J* = 6.54 Hz, 1H, Ar**H**), 7.53 (d, *J* = 7.13 Hz, 1H, Ar**H**), 7.51 (d, *J* = 6.26 Hz, 1H, Ar**H**), 7.47 (t, *J* = 6.98 Hz, 1H, Ar**H**), 7.24 (s, 1H, Ar**H**), 7.01 (dd, *J* = 8.71, 2.17 Hz, 1H, Ar**H**), 6.83 (d, *J* = 2.09 Hz, 1H, Ar**H**), 4.00 (t, *J* = 7.23 Hz, 2H, -NC**H**_2_-), 3.91 (s, 3H, -OC**H**_3_), 1.79 (quint, *J* = 7.26 Hz, 2H, -CH_2_C**H**_**2**_CH_2_-), 1.33–1.23 (m, 4H, -C**H**_2_C**H**_**2**_CH_3_), 0.86 (t, *J* = 6.89 Hz, 3H, -C**H**_**2**_CH_3_) ppm; ^13^C NMR (150.9 MHz, CDCl_3_, 25 °C): *δ* 192.02 (-**C** = O), 157.47 (Ar), 139.17 (Ar), 138.08 (Ar), 137.43 (Ar), 133.83 (Ar), 130.93 (Ar), 130.01 (Ar), 128.24 (Ar), 126.81 (Ar), 126.36 (Ar), 126.12 (Ar), 125.92 (Ar), 124.62 (Ar), 123.71 (Ar), 121.21 (Ar), 117.70 (Ar), 111.82 (Ar), 94.17 (Ar), 55.89 (-O**C**H_3_), 47.16 (-N**C**H_2_-), 29.39 (-NCH_2_**C**H_2_-), 28.98 (-CH_2_**C**H_2_CH_2_-), 22.27 (-**C**H_2_CH_3_), 13.97 (-CH_2_**C**H_3_) ppm. HRMS (EI) calcd for C_25_H_25_NO_2_ [M]⁺· 371.1885, found 371.1885. HPLC purity: 96.88%; retention time: 12.8 min.

#### Compound 5

To a solution of NaH (50 mg, 1.25 mmol, 60% in mineral oil) in dry DMF 4 mL, was added compound **9** (125 mg, 0.415 mmol) and the resulting solution was stirred at rt for 30 min. Then, 1-bromopentane (78 *µ*L, 0.63 mmol, 1.5 equiv) was added and the resulting solution was stirred at 65 °C for 3 h. The reaction was then quenched with sat. NH_4_Cl (10 mL) and stirred for 20 min and extracted with EtOAc (3 × 20 mL). Combined organics were dried over MgSO_4_, solvent evaporated under vacuo and chromatographed to give compound **5** (123 mg, 78%) as a colourless waxy solid.

*R*_f_ = 0.35 (20% EtOAc in hexanes); ^1^H NMR (600 MHz, CDCl_3_) *δ* (ppm) 8.19 (d, *J* = 8.48 Hz, 1H, Ar**H**), 8.02 (d, *J* = 2.46 Hz, 1H, Ar**H**), 7.97 (d, *J* = 8.24 Hz, 1H, Ar**H**), 7.91 (d, *J* = 8.02 Hz, 1H, Ar**H**), 7.65 (dd, *J* = 6.93, 1.07 Hz, 1H, Ar**H**), 7.54–7.50 (m, 2H, Ar**H**), 7.48–7.46 (m, 1H, Ar**H**), 7.27 (d, *J* = 8.08 Hz, 1H, Ar**H**), 6.99 (dd, *J* = 8.82, 2.48 Hz, 1H, Ar**H**), 4.02 (t, *J* = 7.33 Hz, 2H, -NC**H**_2_-), 3.93 (s, 3H, -OC**H**_3_), 1.79 (quint, *J* = 7.42 Hz, 2H, -CH_2_C**H**_**2**_CH_2_-), 1.32–1.21 (m, 4H, -C**H**_2_C**H**_**2**_CH_3_), 0.8 (t, *J* = 7.07 Hz, 3H, -C**H**_**2**_CH_3_) ppm; ^13^C NMR (150.9 MHz, CDCl_3_, 25 °C): *δ* 192.08 (**C** = O), 156.76 (Ar), 139.26 (Ar), 138.00 (Ar), 133.81 (Ar), 132.00 (Ar), 130.88 (Ar), 129.94 (Ar), 128.24 (Ar), 127.91 (Ar), 126.78 (Ar), 126.35 (Ar), 126.09 (Ar), 125.83 (Ar), 124.65 (Ar), 117.23 (Ar), 114.23 (Ar), 110.95 (Ar), 104.04 (Ar), 55.91 (-O**C**H_3_), 47.41 (-N**C**H_2_-), 29.58 (-NCH_2_**C**H_2_-), 28.94 (-CH_2_**C**H_2_CH_2_-), 22.22 (-**C**H_2_CH_3_), 13.94 (-CH_2_**C**H_3_) ppm. HRMS (EI) calcd for C_25_H_25_NO_2_ [M]⁺· 371.1885, found 371.1884. HPLC purity: 99.69%; retention time: 13.1 min.

### Animals

Four-week-old female C57BL/6 mice were purchased from Hyochang Science, Korea. All mice were housed under controlled environmental conditions with a 12-h light/dark cycle, a temperature of 22 ± 2 °C, and a humidity of 50 ± 10%. The animals were provided with ad libitum access to a standard laboratory diet and sterilized water. All animal experiments and procedures were performed in accordance with the Guide for the Care and Use of Laboratory Animals (8th edition, National Research Council, 2011). The study protocol was approved by the Institutional Animal Care and Use Committee (IACUC) of Daegu Catholic University (approval number: CUD-2025-043).

### Drug preparation and administration

**JWH-018** (**1**) and its methoxy derivatives (**4** and **5**) were dissolved in a vehicle composed of 5% ethanol, 5% Tween 80, and 90% phosphate-buffered saline (PBS). Stock solutions were initially prepared at a concentration of 10 mg/kg based on the average body weight of the mice. Subsequent doses of 1 mg/kg and 3 mg/kg were obtained by serial dilution with the same vehicle. The dose range (1–10 mg/kg) was determined based on literature precedent, as **JWH-018** (**1**) has been reported to induce significant, dose-dependent cannabinoid-like effects in the mouse tetrad assay starting at 3 mg/kg (i.p.). Preliminary pilot studies in our laboratory further confirmed that 3 mg/kg of **JWH-018** (**1**) serves as a reliable benchmark for inducing measurable behavioral responses across all tetrad parameters. All compounds and the vehicle control were administered via intraperitoneal (i.p.) injection in a fixed volume of 200 µL per mouse.

### Catalepsy assessment

Catalepsy was assessed 15 min after injection using the **bar test**. The mouse’s forepaws were placed on a horizontal bar positioned at a height of 4 cm from the floor. The duration for which the mouse maintained this imposed posture was recorded. The test ended when the mouse removed its paws from the bar or after a maximum cut-off time of 30 s.

### Hypothermia assessment

Rectal temperature was measured 30 min post-injection to assess hypothermia. A calibrated digital rectal thermometer was gently inserted into the rectum to a depth of approximately 2 cm. The stable temperature reading was recorded.

### Analgesia assessment

Antinociceptive activity (analgesia) was evaluated 45 min after injection using the **hot plate test**. The mouse was placed on a hot plate maintained at a constant temperature of **55 °C**. The latency to exhibit nociceptive responses, such as hind paw licking, shaking, or jumping, was measured. A cut-off time of 30 s was applied to prevent tissue damage.

### Immobility (locomotor activity) assessment

Spontaneous locomotor activity was assessed 60 min post-injection using an open field arena (40 × 40 × 40 cm). The floor of the arena was divided into equal square quadrants (cells). Each mouse was placed in the center of the arena, and its activity was monitored for 5 min. Locomotor activity was quantified by counting the number of cells crossed (grid crossings), defined as the mouse moving all four paws into a new cell. Additionally, the total duration of immobility (defined as the complete absence of voluntary movement except for respiration) was recorded during the test period. The arena was thoroughly cleaned with 70% ethanol between each trial to eliminate olfactory cues.

### Statistical analysis

Statistical analyses were performed using GraphPad Prism 10.0. All data are presented as mean ± standard error of the mean (SEM). To determine the significance of differences between multiple experimental groups, a one-way analysis of variance (ANOVA) was conducted. Following the ANOVA, Tukey’s multiple comparison test was applied to identify specific differences between pairs of groups. For all experiments involving animal subjects, a sample size of *N* = 5 mice per group was utilized to ensure statistical power. A p-value of less than 0.05 (*p* < 0.05) was considered statistically significant.

## Supplementary Information

Supplementary Information.

## Data Availability

All data generated or analysed during this study are included in this published article and its supplementary information files.
