# Supplementary material for: In vivo evaluation of synthetic cannabinoid JWH-018 derivatives as potential new psychoactive substances
Source: Sci Rep. 2026 May 11;16:21528. doi: 10.1038/s41598-026-51639-z (PMC13350963; doi:10.1038/s41598-026-51639-z)
Supplement: Supplementary file 1 — Supplementary Information. [file 41598_2026_51639_MOESM1_ESM.docx]

***In Vivo* Evaluation of Synthetic Cannabinoid JWH-018 Derivatives as Potential New Psychoactive Substances**

Salman Khan^†^, Minseo Baek^†^, Jeong-Hoon Jang*, and In-Soo Myeong*

*College of Pharmacy, Daegu Catholic University, Gyeongsan, Gyeongbuk 38430, Republic of Korea*

^†^ Both authors contributed equally to this work.

**Table of Content**

| **Figure S1.** ^1^H NMR spectroscopic characterization of **1** | … SI-2 |
| --- | --- |
| **Figure S2.** ^13^C NMR spectroscopic characterization of **1** | … SI-3 |
| **Figure S3.** ^1^H NMR spectroscopic characterization of **7** | … SI-4 |
| **Figure S4.** ^13^C NMR spectroscopic characterization of **7** | … SI-5 |
| **Figure S5.** ^1^H NMR spectroscopic characterization of **4** | … SI-6 |
| **Figure S6.** ^13^C NMR spectroscopic characterization of **4** | … SI-7 |
| **Figure S7.** ^1^H NMR spectroscopic characterization of **9** | … SI-8 |
| **Figure S8.** ^13^C NMR spectroscopic characterization of **9** | … SI-9 |
| **Figure S9.** ^1^H NMR spectroscopic characterization of **5** | … SI-10 |
| **Figure S10.** ^13^C NMR spectroscopic characterization of **5** | … SI-11 |
| **Figure S11.** HPLC chromatogram of **1** | … SI-12 |
| **Figure S12.** HPLC chromatogram of **4** | … SI-13 |
| **Figure S13.** HPLC chromatogram of **5** | … SI-14 |

***
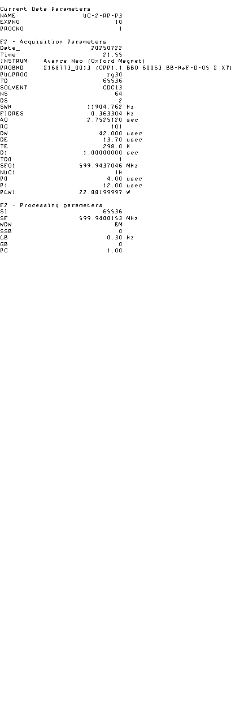

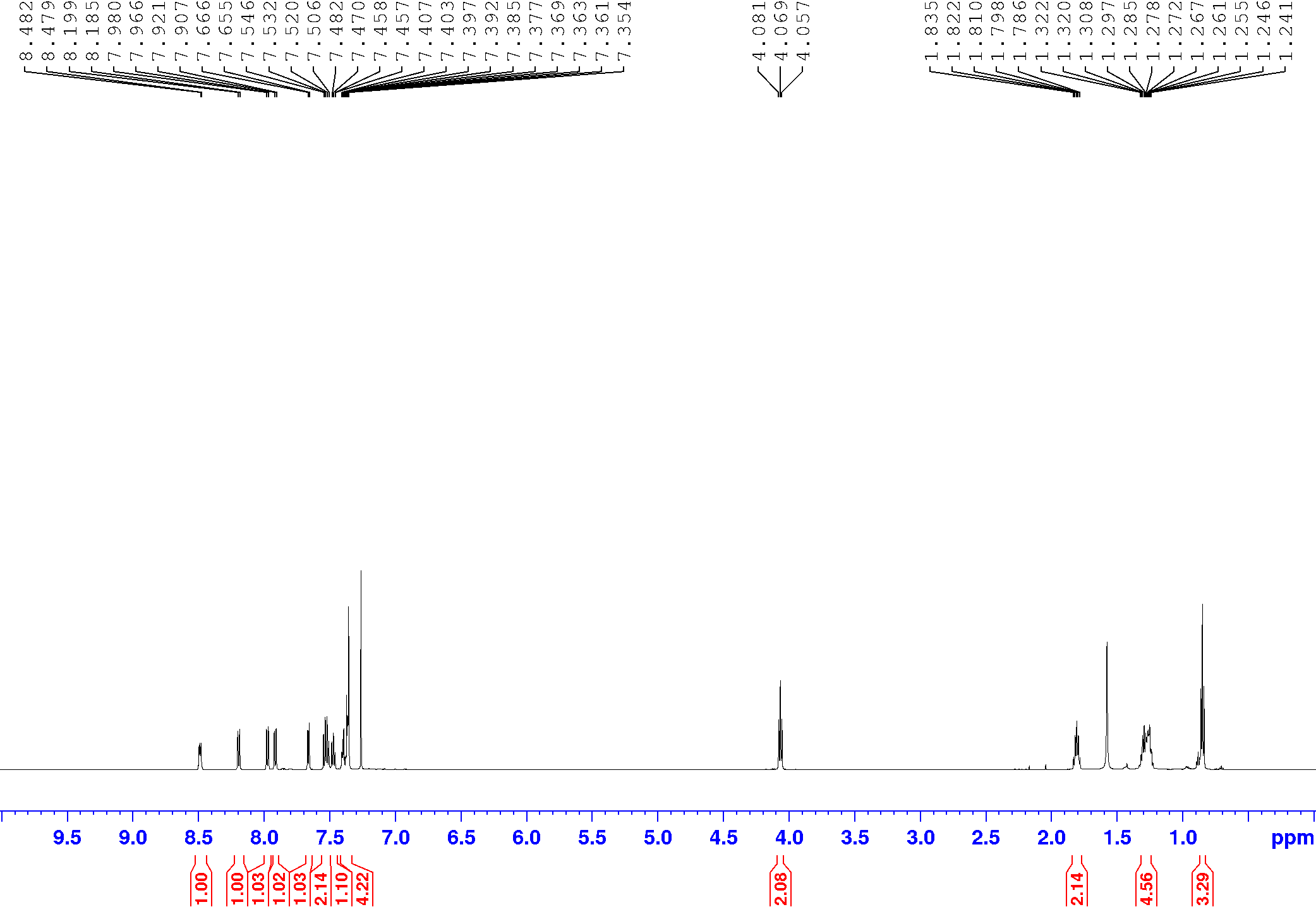
***

**Figure S1.** ^1^H NMR of compound **1**

***
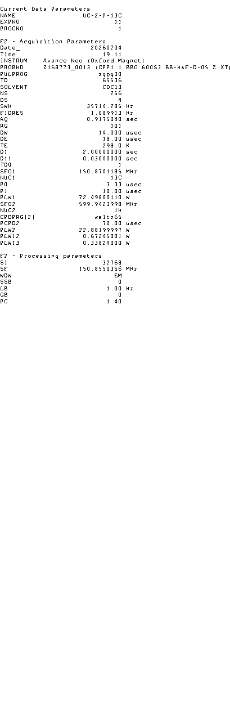
***
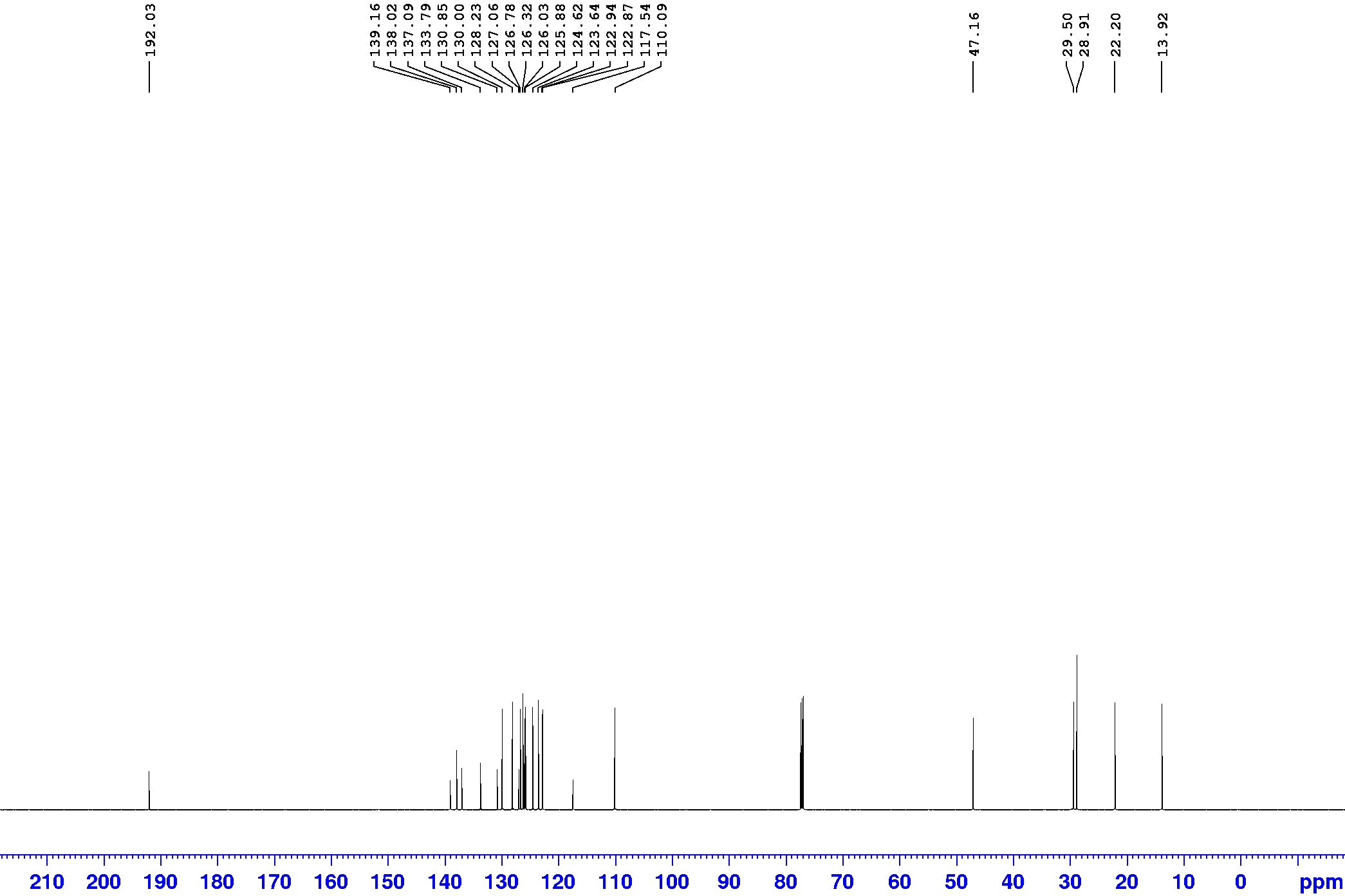


**Figure S2.** ^13^C NMR of compound **1**

***
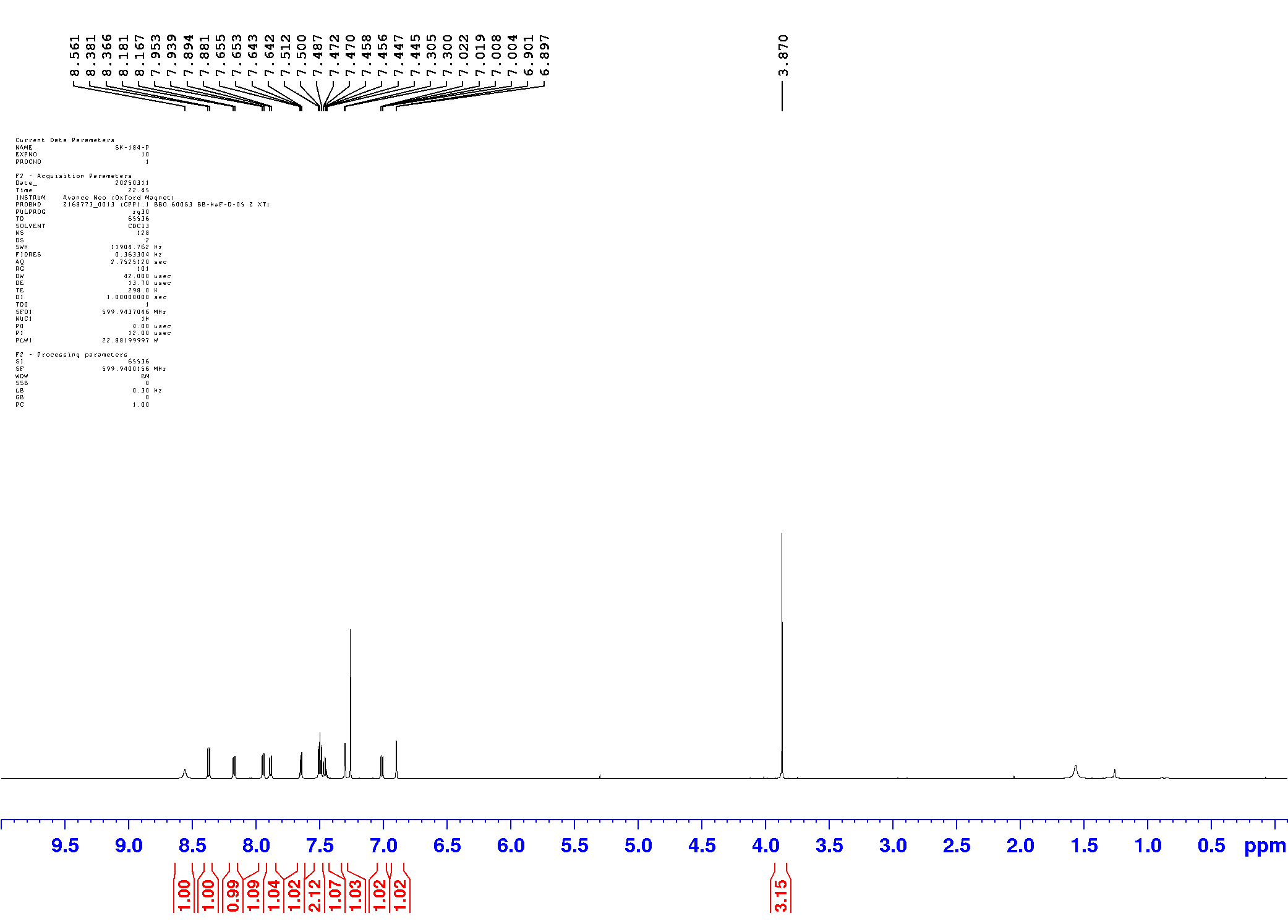
***

**Figure S3.** ^1^H NMR of compound **7**


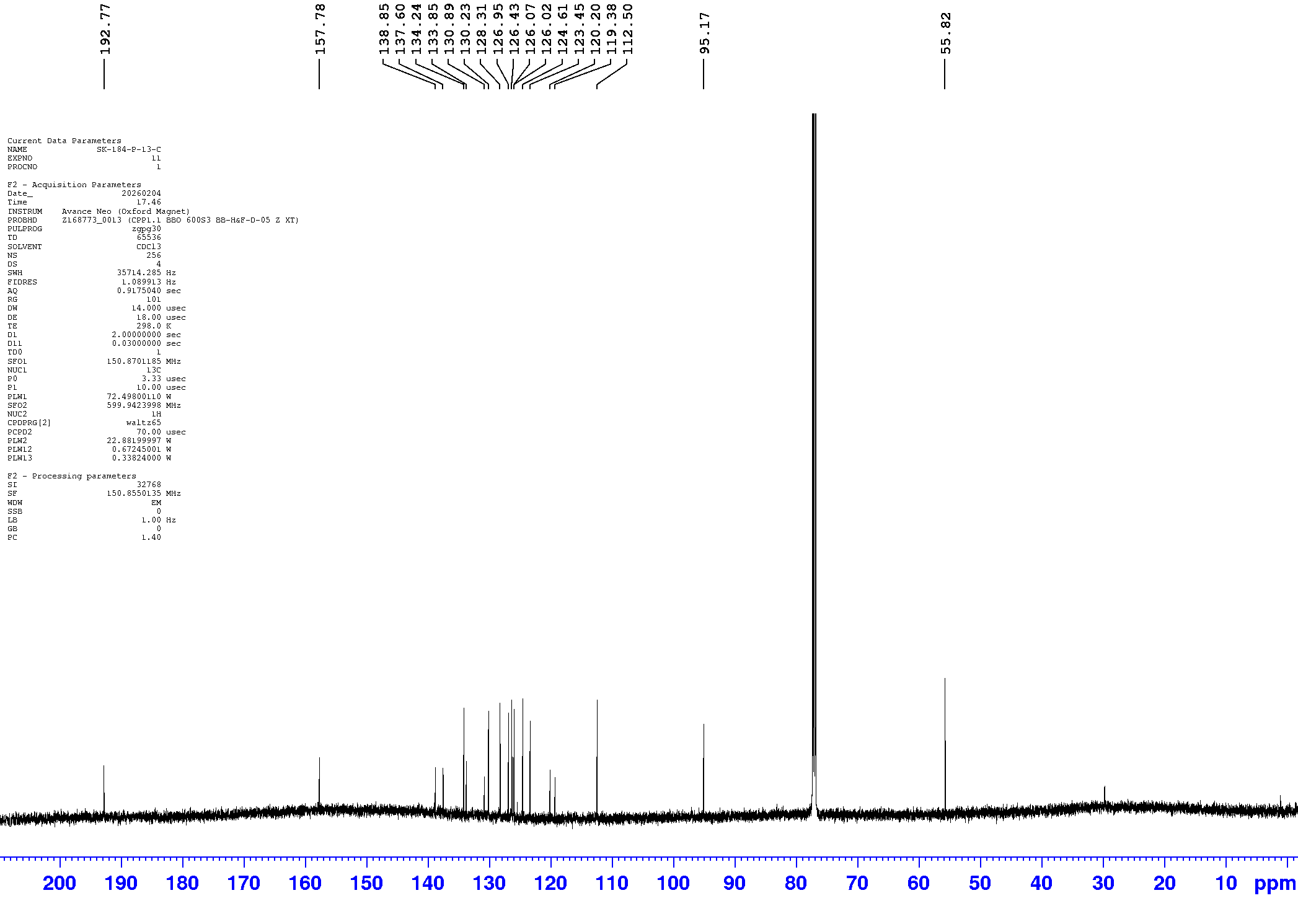


**Figure S4.** ^13^C NMR of compound **7**

***
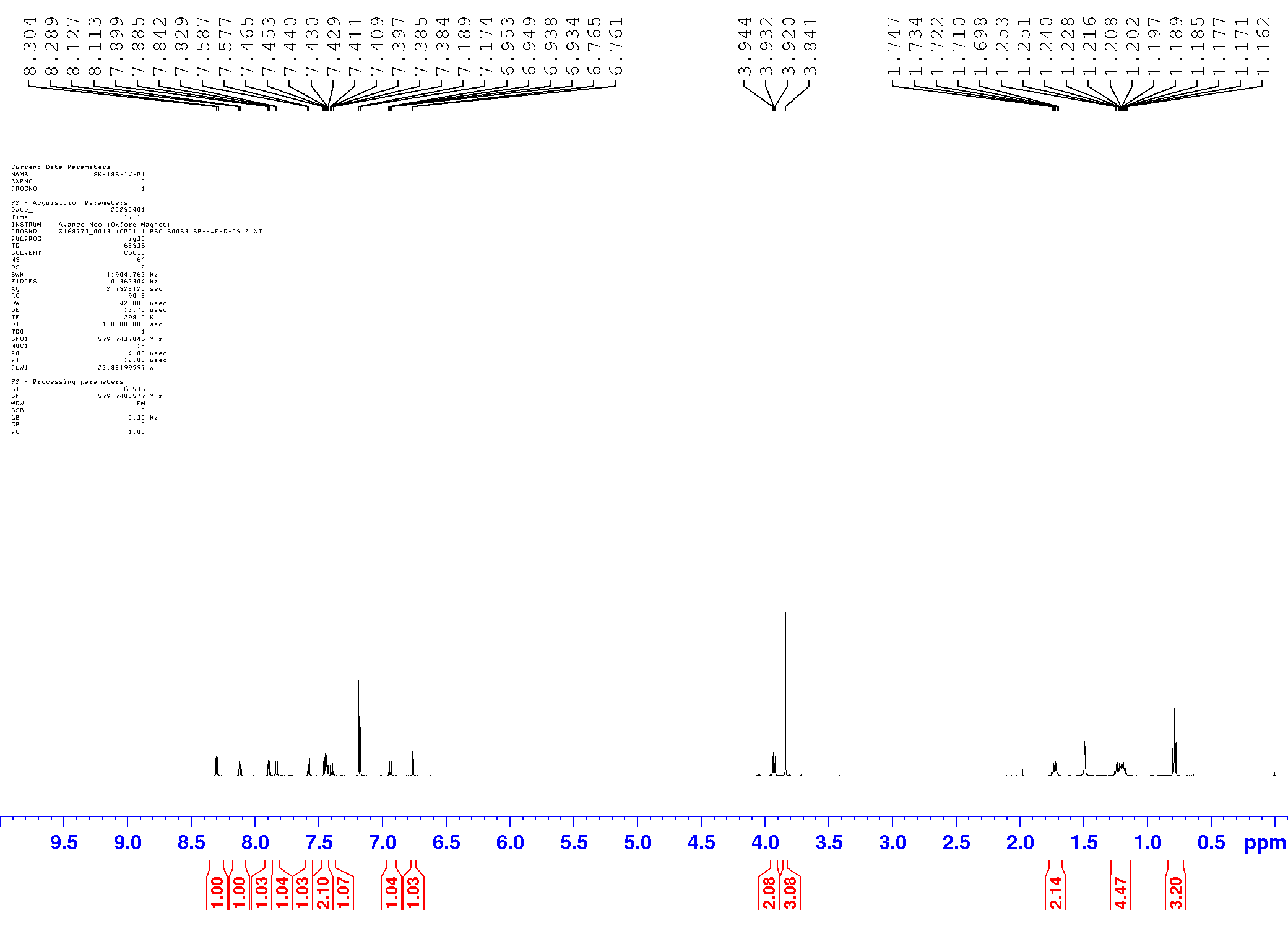
***

**Figure S5.** ^1^H NMR of compound **4**

******
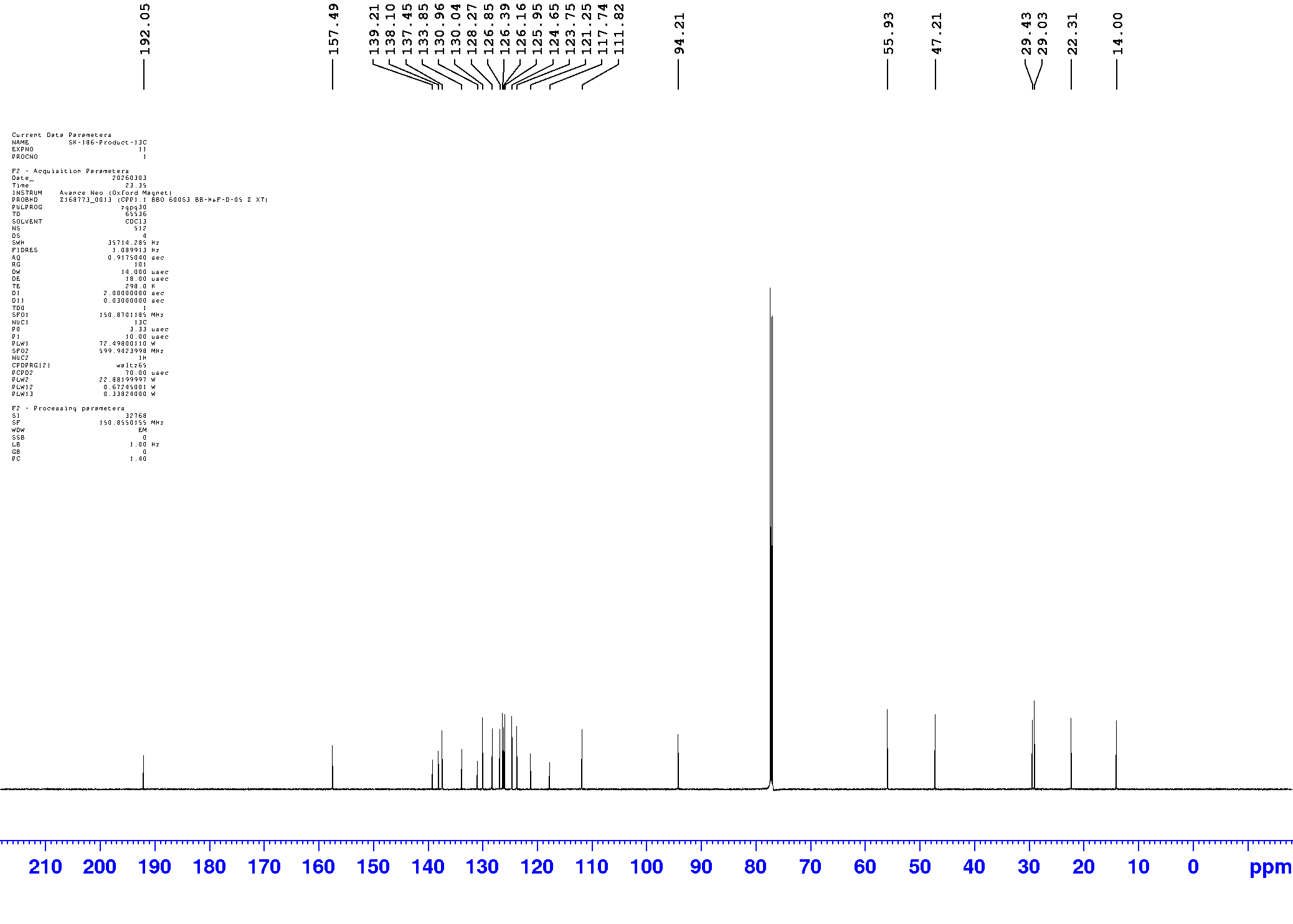


**Figure S6.** ^13^C NMR of compound **4**

***
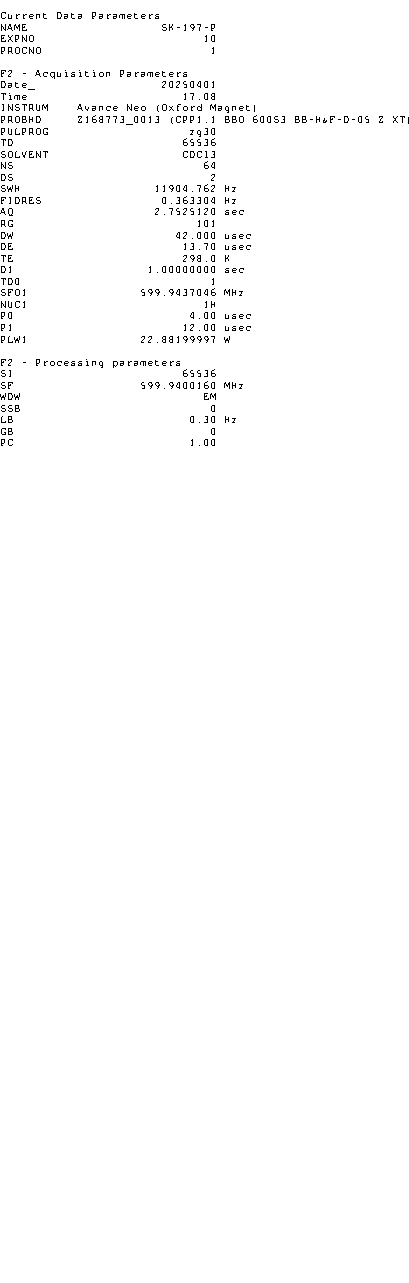

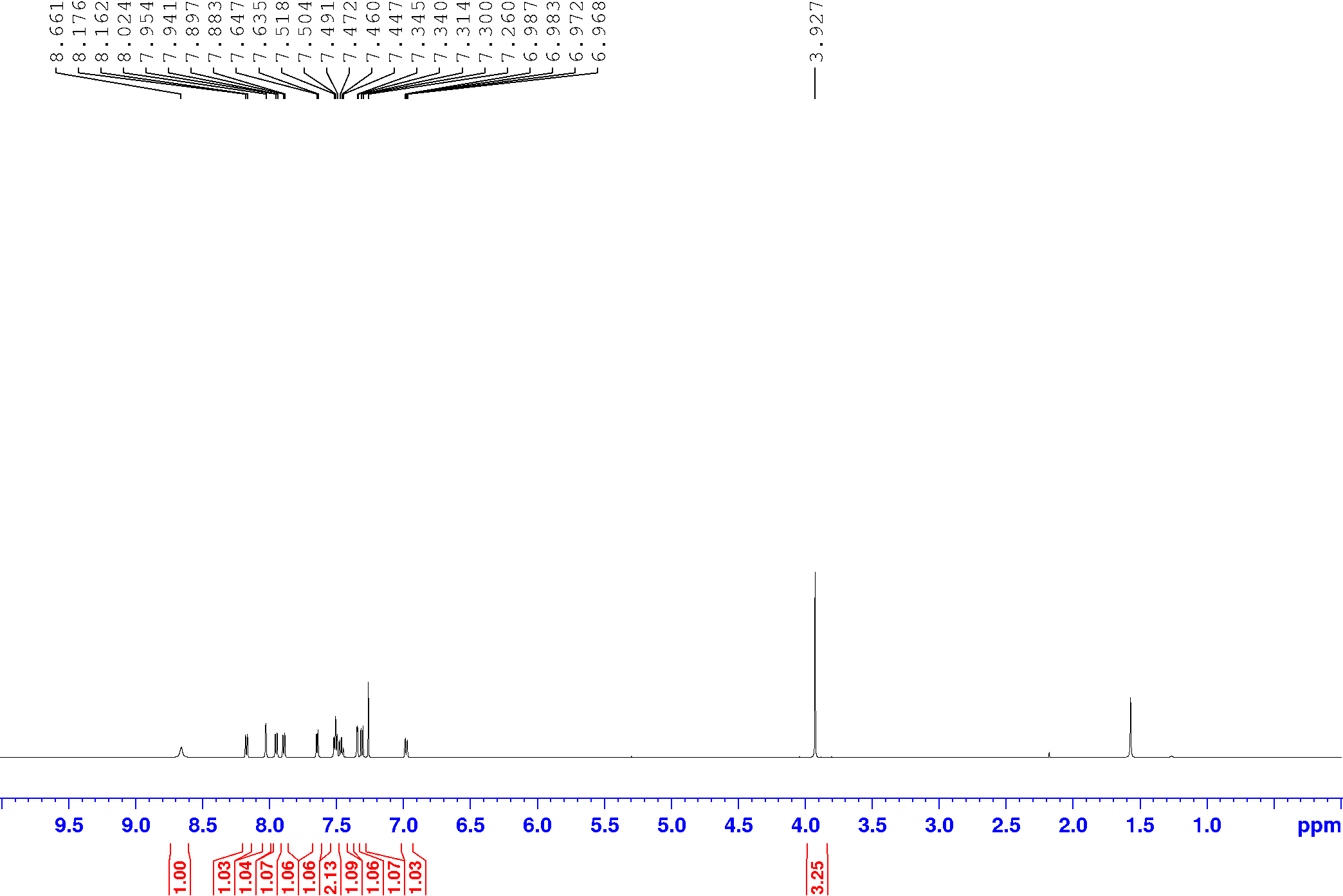
***

**Figure S7.** ^1^H NMR of compound **9**

**
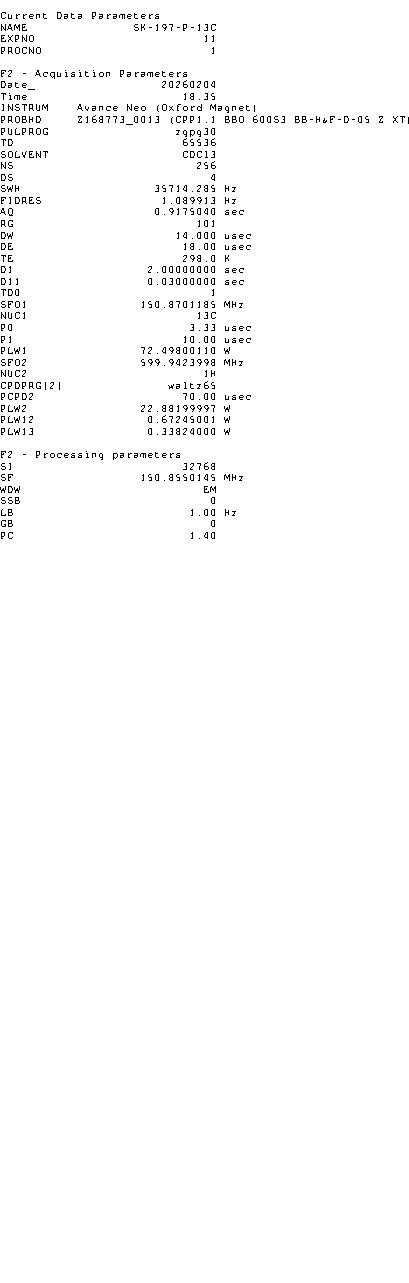
**
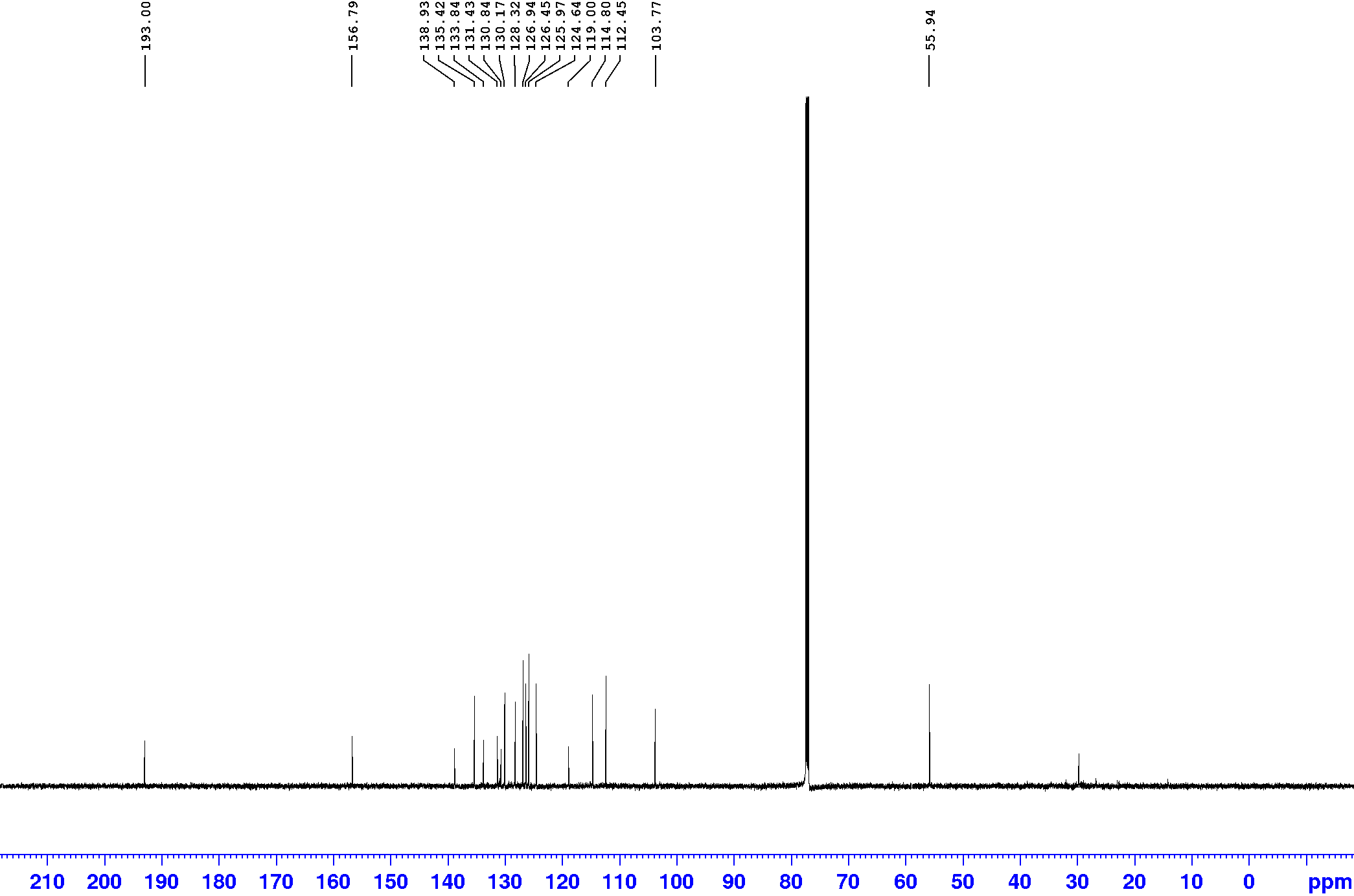


**Figure S8.** ^13^C NMR of compound **9**


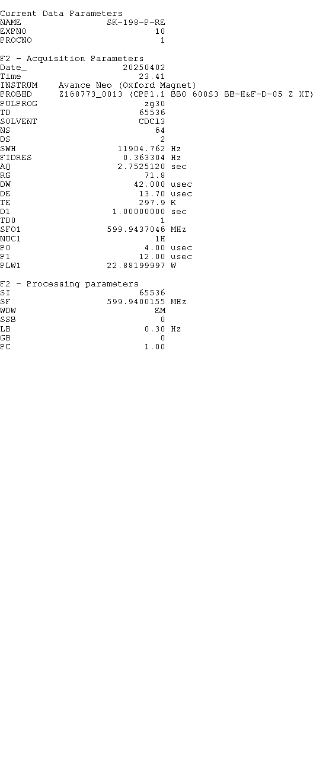

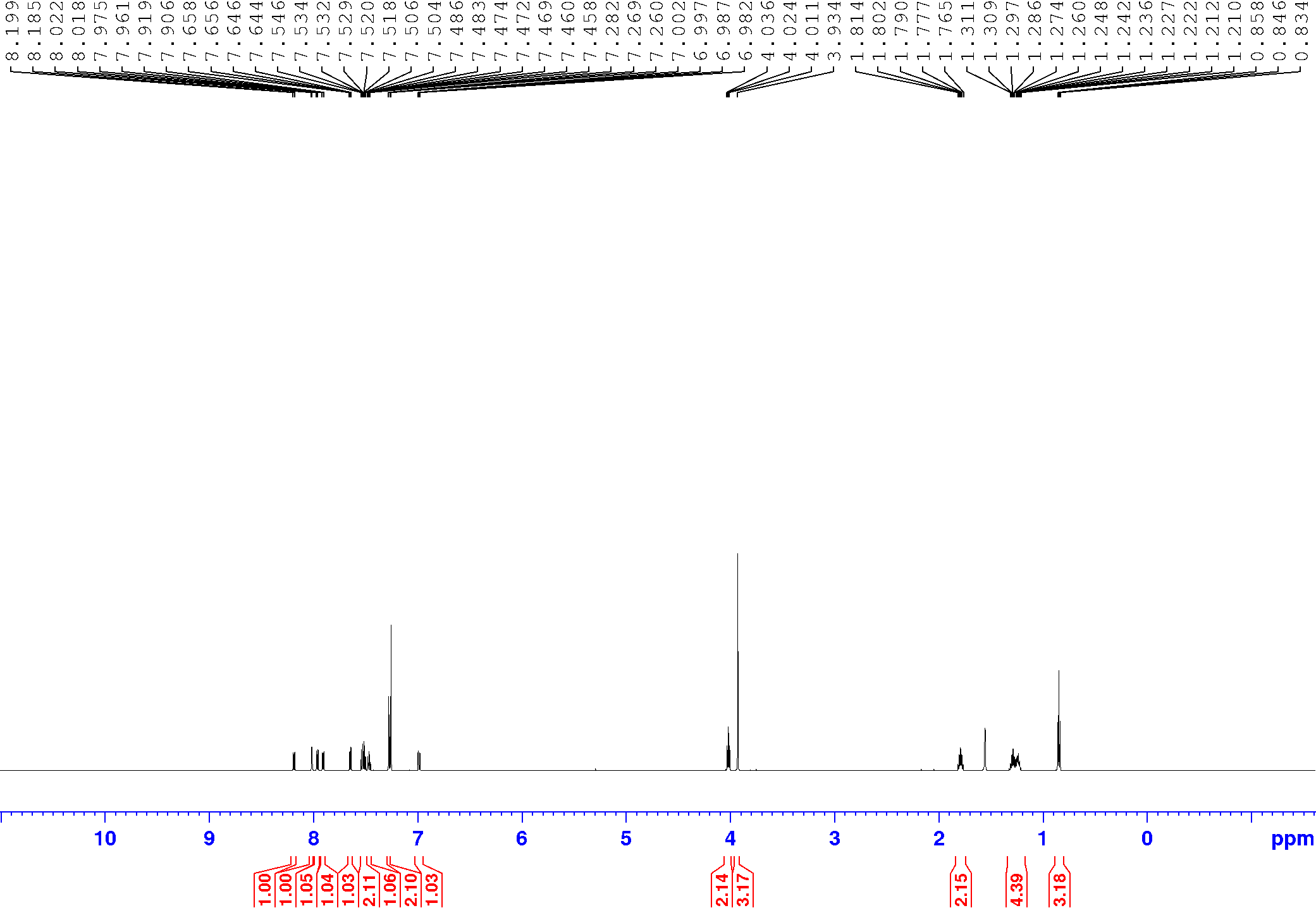


**Figure S9.** ^1^H NMR of compound **5**

**
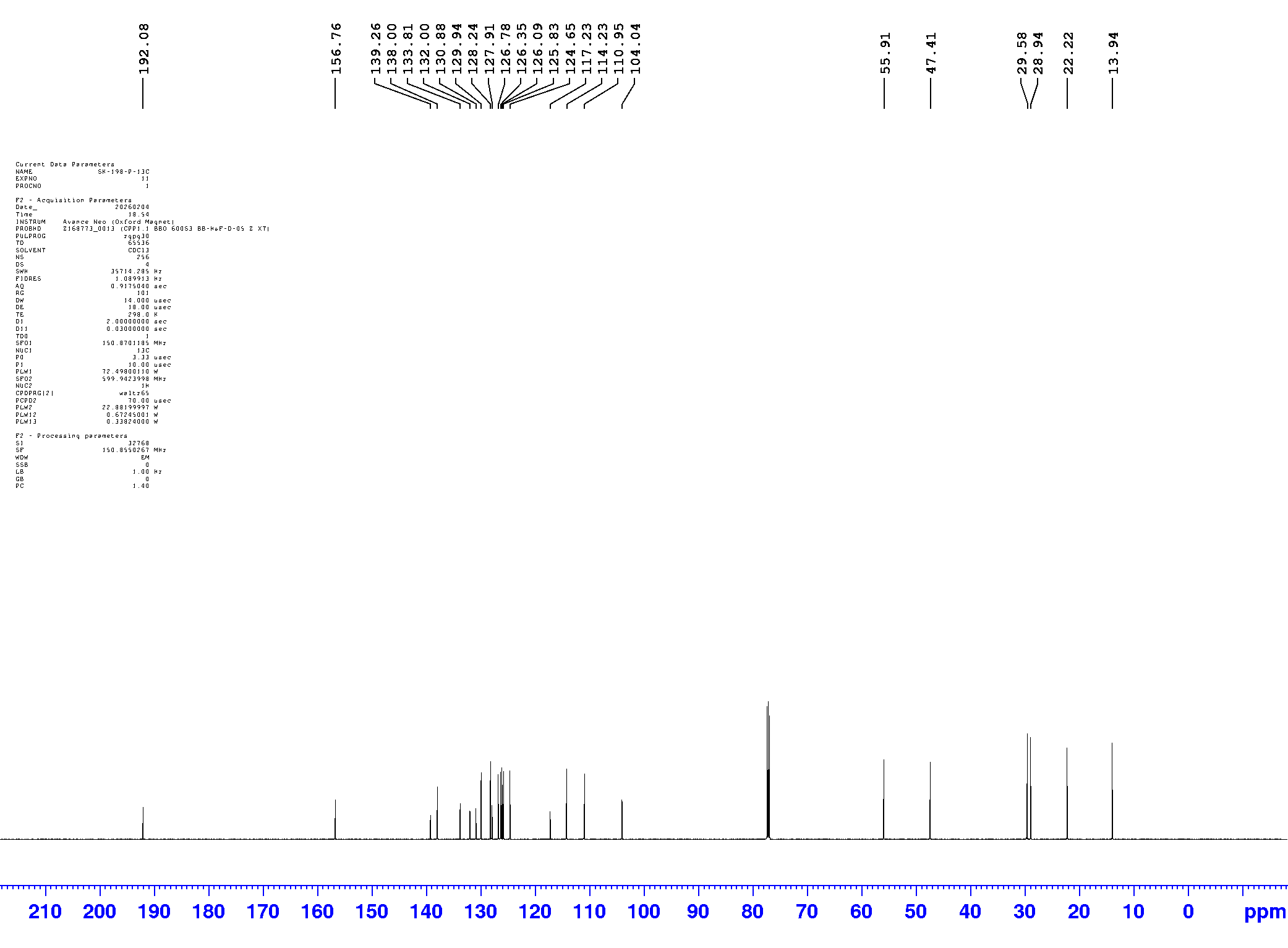
**

**Figure S10.** ^13^C NMR of compound **5**


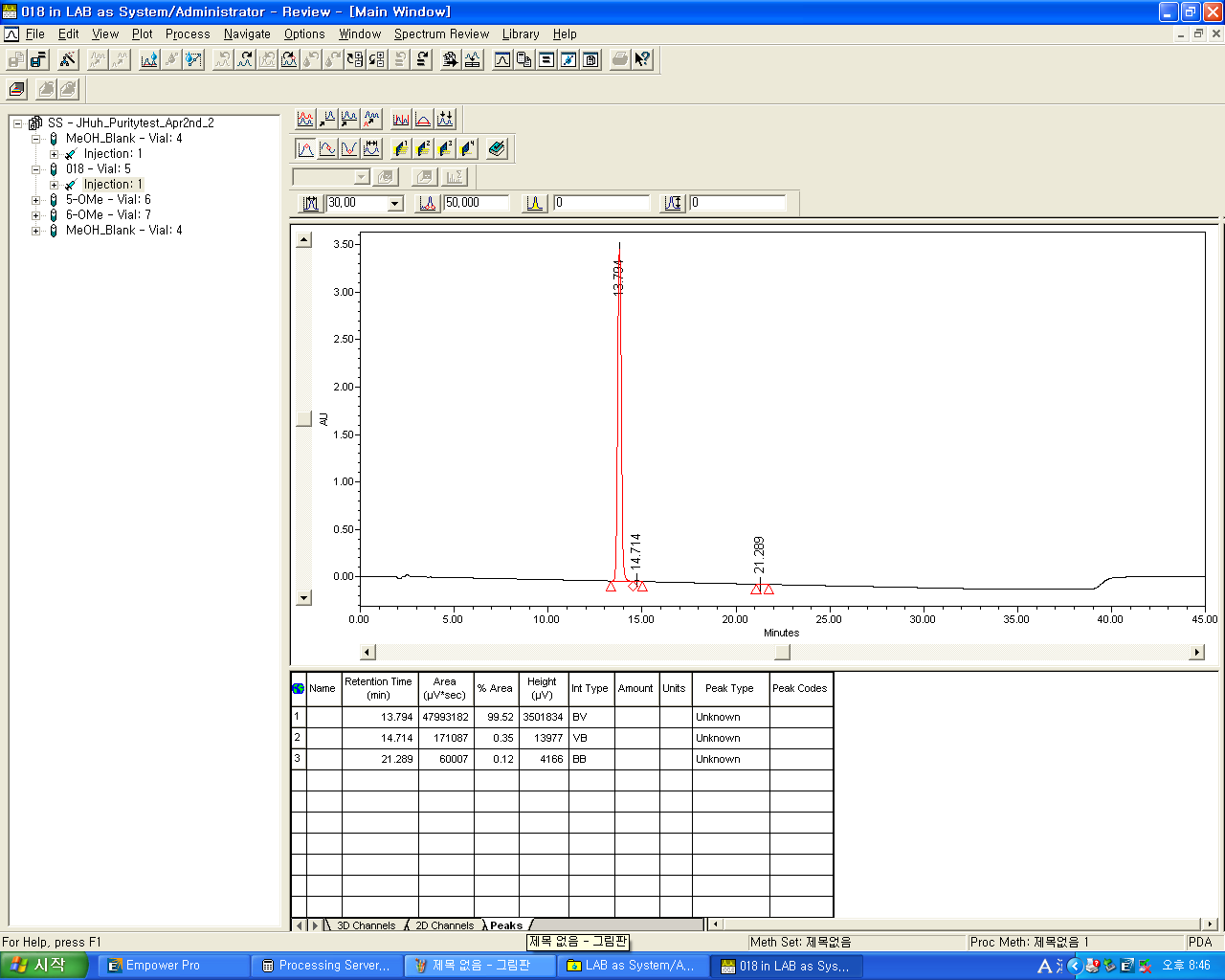


**Figure S11.** HPLC data for **1**


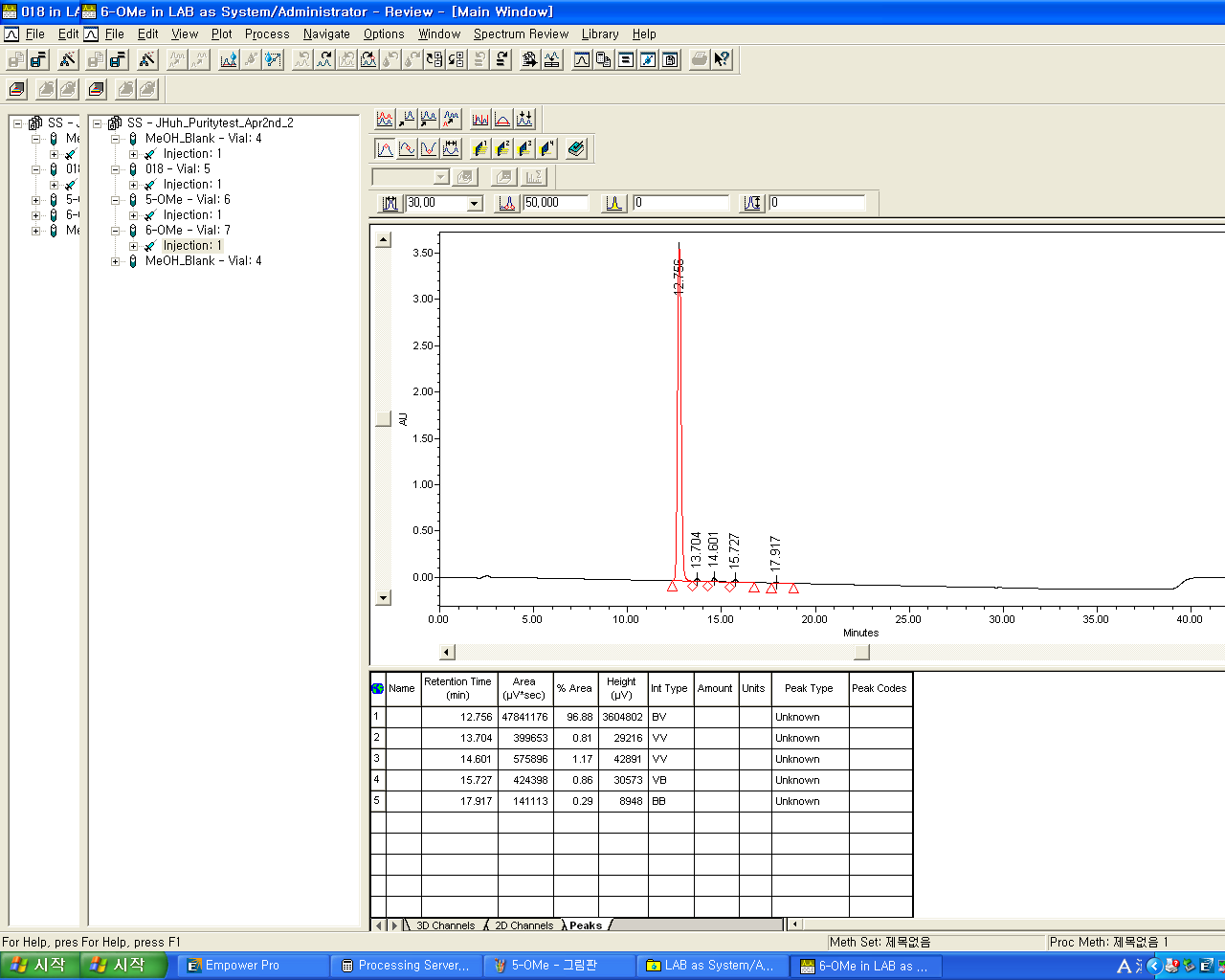


**Figure S12.** HPLC data for **4**


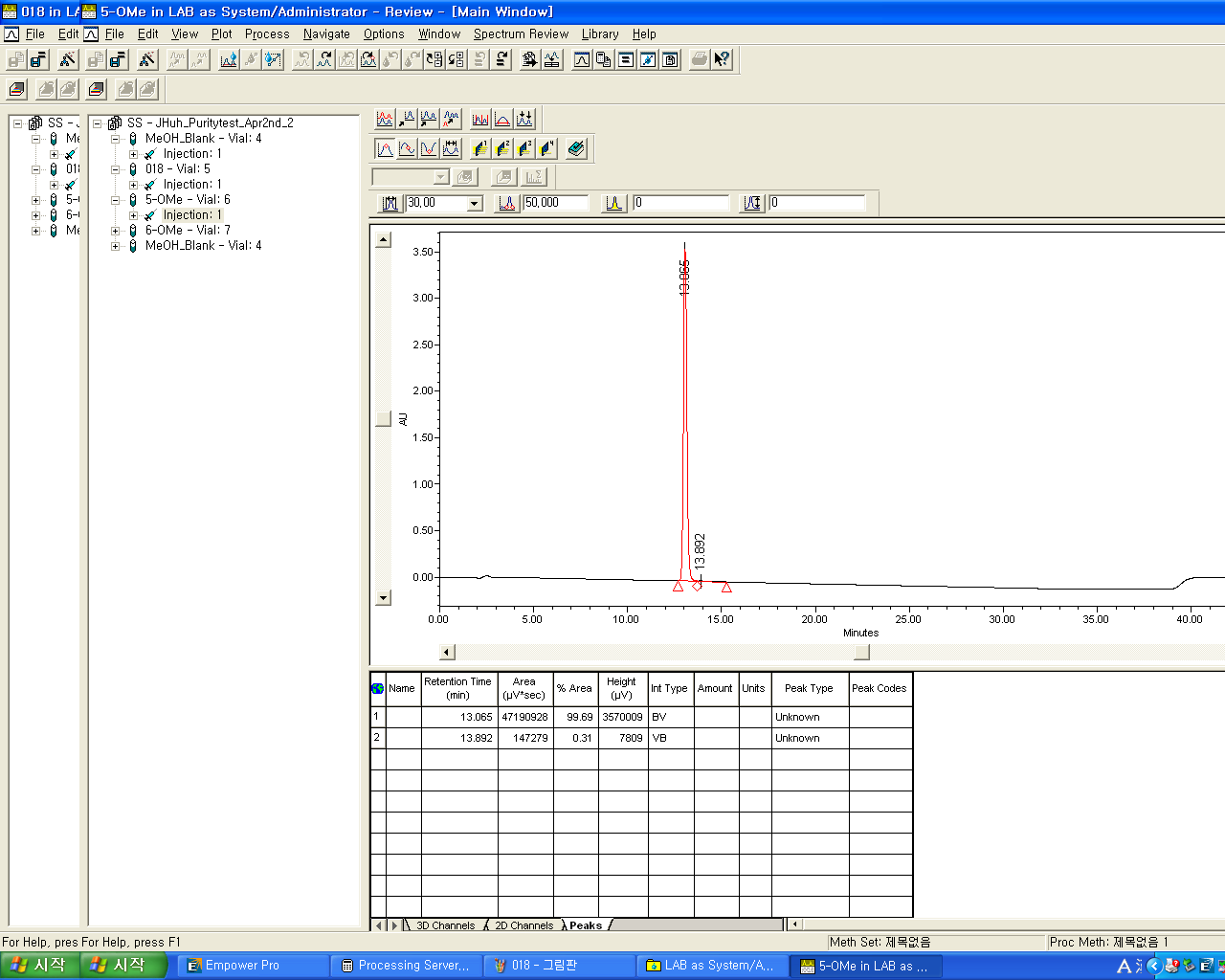


**Figure S13.** HPLC data for **5**
